# Supplementary material for: Transposon accumulation at xenobiotic gene family loci in aphids
Source: Genome Res. 2023 Oct;33(10):1718–33. doi: 10.1101/gr.277820.123 (PMC10691553; doi:10.1101/gr.277820.123)
Supplement: Supplement 1 [file Supplemental_Figure_S1.pdf]

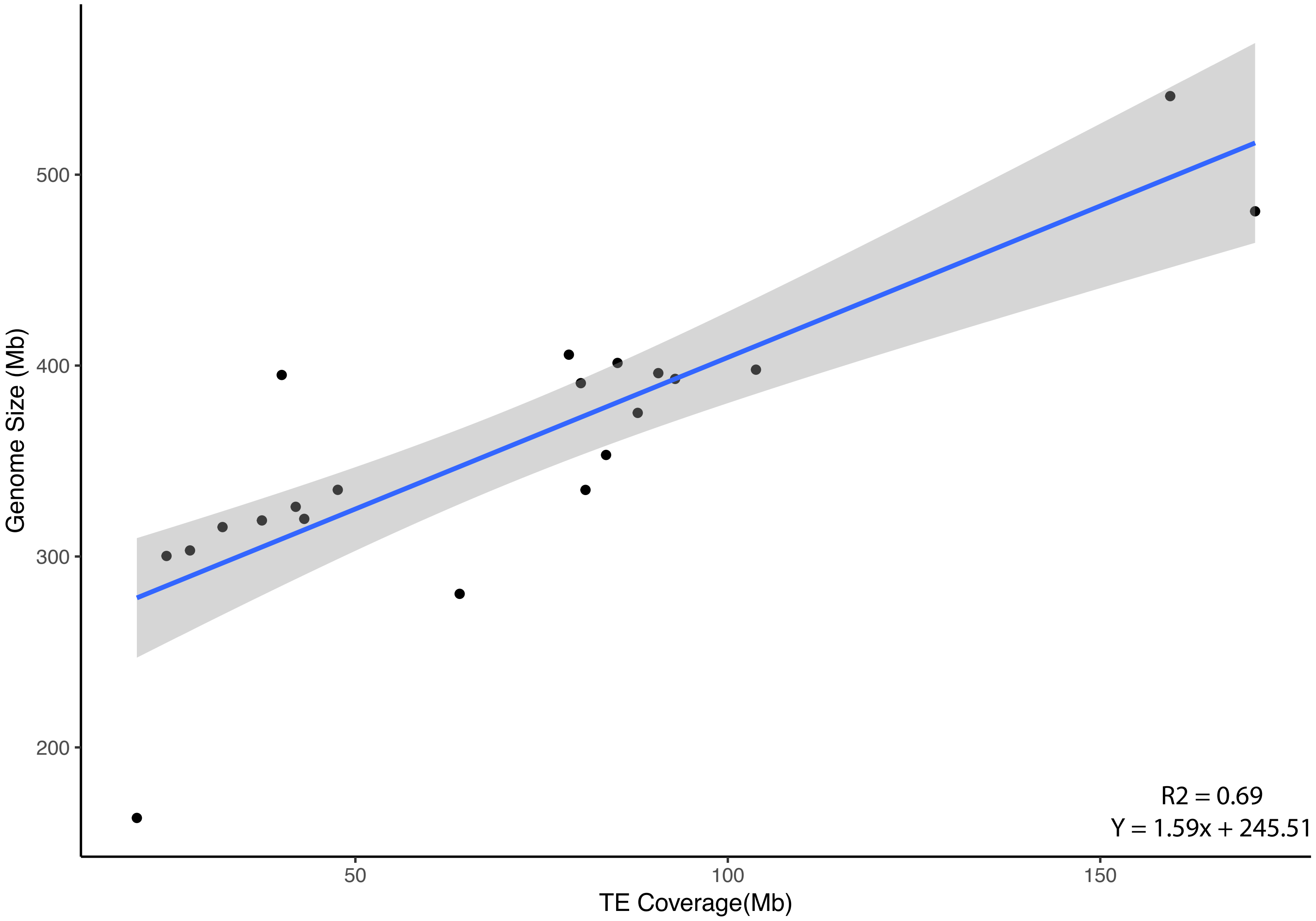

Supplemental Figure S1. The relationship between TE content and genome size in aphids.
Blue line indicates the relationship calculated using linear regression. Grey area indicates 95% confidence intervals.
