# Supplementary material for: Transposon accumulation at xenobiotic gene family loci in aphids
Source: Genome Res. 2023 Oct;33(10):1718–33. doi: 10.1101/gr.277820.123 (PMC10691553; doi:10.1101/gr.277820.123)
Supplement: Supplement 2 [file Supplemental_Figure_S2.pdf]

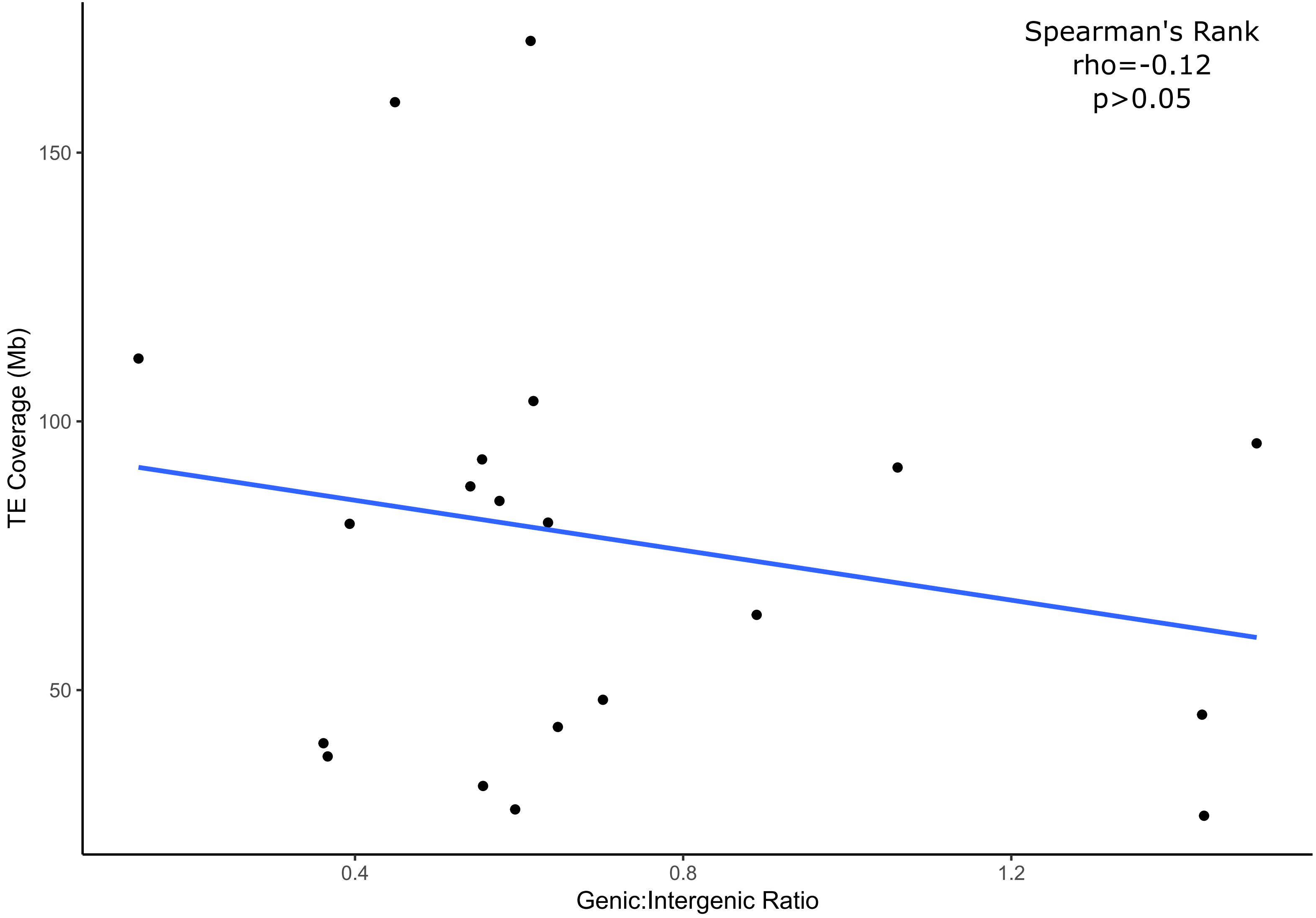


Supplemental Figure S2. Correlation between genic:intergenic base pairs ratio and TE content in aphids.
