# Supplementary material for: Transposon accumulation at xenobiotic gene family loci in aphids
Source: Genome Res. 2023 Oct;33(10):1718–33. doi: 10.1101/gr.277820.123 (PMC10691553; doi:10.1101/gr.277820.123)
Supplement: Supplement 3 [file Supplemental_Figure_S3.pdf]

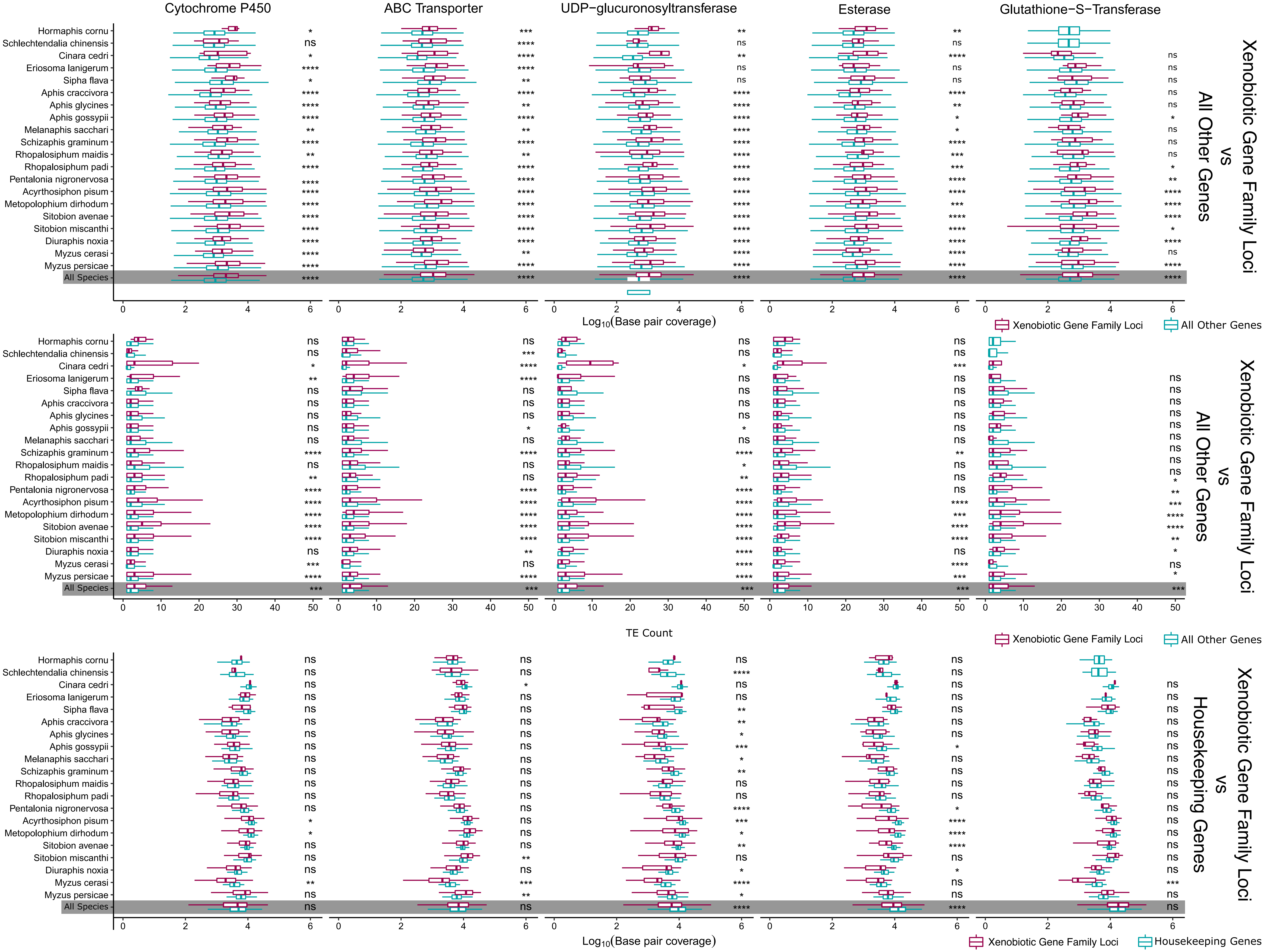

Supplemental Figure S3. TE abundance around xenobiotic gene family loci compared to all other genes and housekeeping genes, split by xenobiotic gene family type.
