# Supplementary material for: Transposon accumulation at xenobiotic gene family loci in aphids
Source: Genome Res. 2023 Oct;33(10):1718–33. doi: 10.1101/gr.277820.123 (PMC10691553; doi:10.1101/gr.277820.123)
Supplement: Supplement 4 [file Supplemental_Figure_S4.pdf]

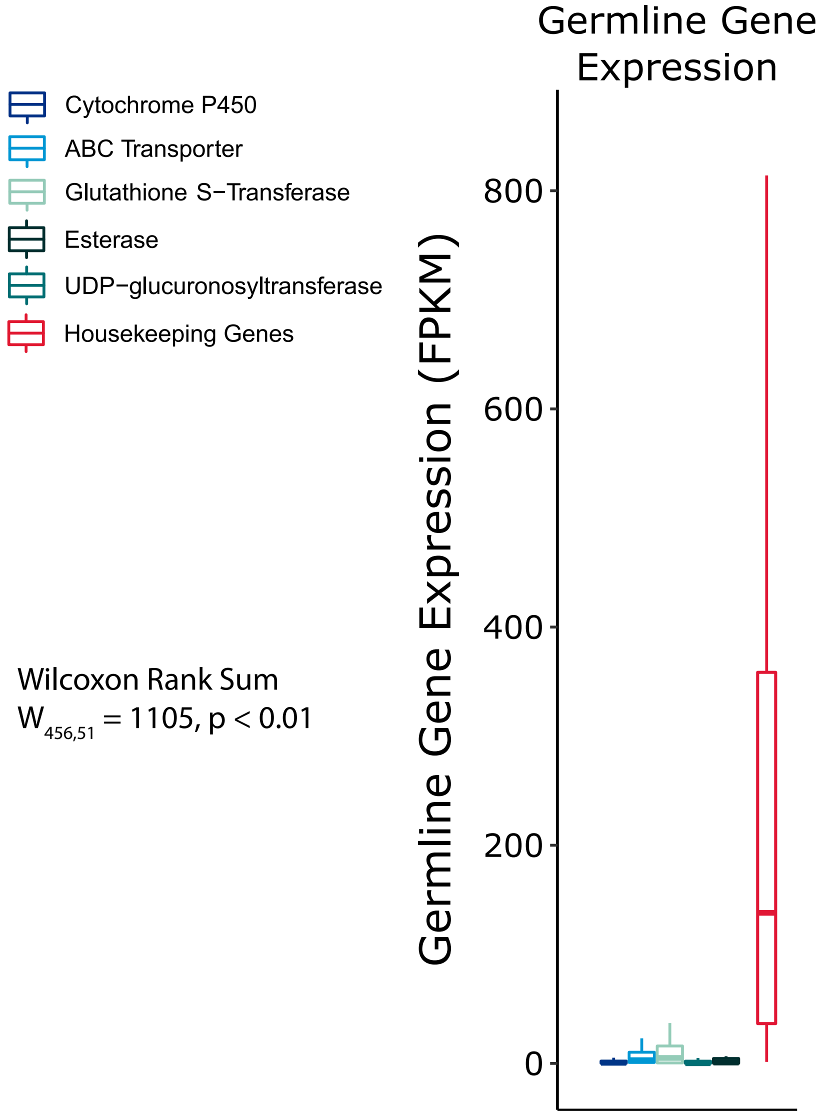

Supplemental Figure S4. Expression of XGFs and housekeeping genes in germline tissues (testis and ovary) in D. melanogaster using data from FlyAtlas2 (Krause et al. 2022). Expression is measured as fragments per kilobase of transcript per million base pairs sequenced (FPKM), to determine expected expression of XGFs in germline cell types. Gene types are indicated by colours in the key.
