# Supplementary material for: Transposon accumulation at xenobiotic gene family loci in aphids
Source: Genome Res. 2023 Oct;33(10):1718–33. doi: 10.1101/gr.277820.123 (PMC10691553; doi:10.1101/gr.277820.123)
Supplement: Supplement 5 [file Supplemental_Figure_S5.pdf]

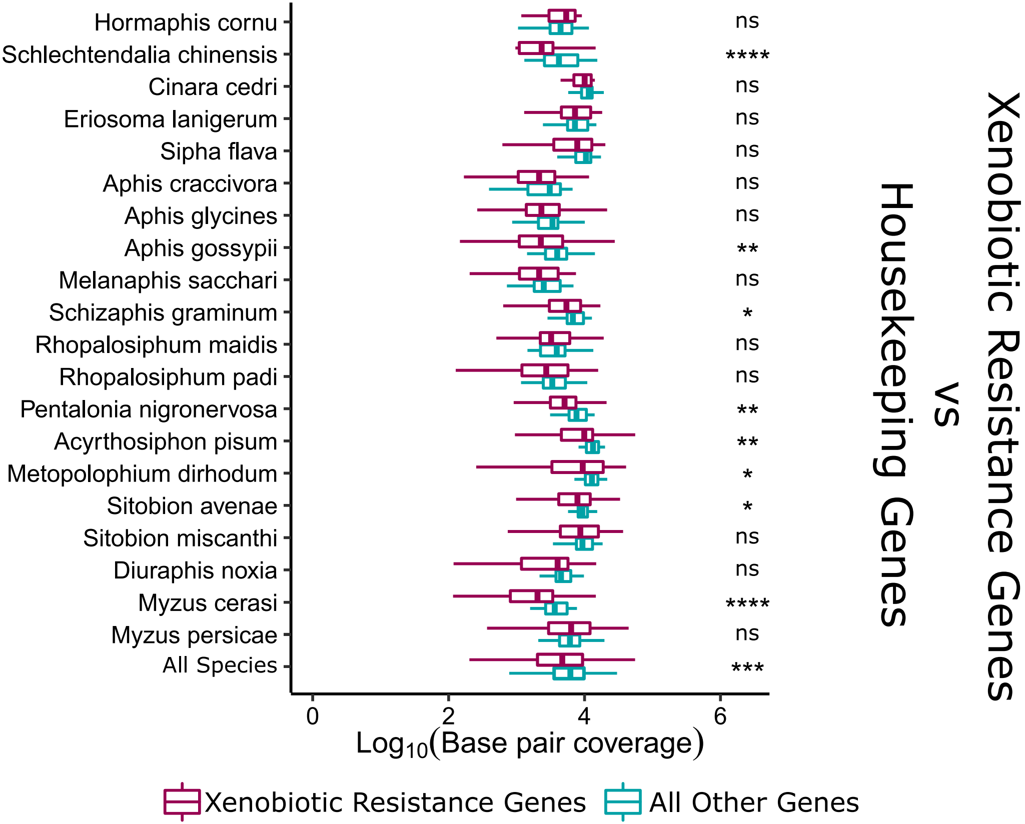

Supplemental Figure S5. TE coverage around xenobiotic gene family loci compared to housekeeping genes.
